# Characterization of Temozolomide Resistance Using a Novel Acquired Resistance Model in Glioblastoma Cell Lines

**DOI:** 10.3390/cancers14092211

**Published:** 2022-04-28

**Authors:** Yuan Zhu, Zhen Chen, Su Na Kim, Chao Gan, Tatsiana Ryl, Michaela Silvia Lesjak, Jan Rodemerk, Rong De Zhong, Karsten Wrede, Philipp Dammann, Ulrich Sure

**Affiliations:** 1 Department of Neurosurgery and Spine Surgery, University Hospital Essen, University of Duisburg-Essen, 45147 Essen, Germany; zhen.chen-neurosurgery@outlook.com (Z.C.); suna.kim@uk-essen.de (S.N.K.); ganchao17@outlook.com (C.G.); tatsiana.ryl@uk-essen.de (T.R.); michaela.s.lesjak@gmail.com (M.S.L.); jan.rodemerk@uk-essen.de (J.R.); zhongrongde@gmail.com (R.D.Z.); karsten.wrede@uk-essen.de (K.W.); philipp.dammann@uk-essen.de (P.D.); ulrich.sure@uk-essen.de (U.S.); 2 Center for Translational Neuro- & Behavioral Sciences (C-TNBS), University of Duisburg-Essen, 45147 Essen, Germany

**Keywords:** glioblastoma, temozolomide resistance, DNA damage, cell proliferation and apoptosis, cell cycle, self-renewal, stemness

## Abstract

**Simple Summary:**

Temozolomide (TMZ) is the first-line drug for chemotherapy of GBM, the most aggressive and incurable brain tumor. Acquired chemoresistance is a hallmark that causes the poor prognosis of GBM. Therefore, understanding the underlying mechanisms by using a proper model becomes emergent. Previous models usually take weeks/months and are often not fully representative of characteristics of TMZ resistance. We established an acute acquired TMZ resistance model using GBM cell lines with different genomic backgrounds. In response to TMZ, the resistant cells showed less susceptibility and sustained regrowth, high clonogenicity, reduced DNA damage accompanied by attenuated MMR, shortened G2/M arrest, uncontrolled DNA replication, and evasion of apoptosis. Moreover, these TMZ resistant cells presented stem cell properties that are critical for chemoresistance. Thus, our model recapitulates all key features of TMZ resistance and is believed to be a promising model to study the underlying mechanisms and define therapeutics for GBM in the future.

**Abstract:**

Temozolomide (TMZ) is the first line of standard therapy in glioblastoma (GBM). However, relapse occurs due to TMZ resistance. We attempted to establish an acquired TMZ resistance model that recapitulates the TMZ resistance phenotype and the relevant gene signature. Two GBM cell lines received two cycles of TMZ (150 µM) treatment for 72 h each. Regrown cells (RG2) were defined as TMZ resistant cells. MTT assay revealed significantly less susceptibility and sustained growth of RG2 compared with parental cells after TMZ challenge. TMZ-induced DNA damage significantly decreased in 53BP1-foci reporter transduced-RG2 cells compared with parental cells, associated with downregulation of *MSH2* and *MSH6*. Flow cytometry revealed reduced G2/M arrest, increased EdU incorporation and suppressed apoptosis in RG2 cells after TMZ treatment. Colony formation and neurosphere assay demonstrated enhanced clonogenicity and neurosphere formation capacity in RG2 cells, accompanied by upregulation of stem markers. Collectively, we established an acute TMZ resistance model that recapitulated key features of TMZ resistance involving impaired mismatch repair, redistribution of cell cycle phases, increased DNA replication, reduced apoptosis and enhanced self-renewal. Therefore, this model may serve as a promising research tool for studying mechanisms of TMZ resistance and for defining therapeutic approaches to GBM in the future.

## 1. Introduction

Glioblastoma (GBM) is the most common and aggressive malignant brain tumor. The current standard of care for GBM consists of gross total surgical resection followed by radiation with concurrent and adjuvant chemotherapy. However, the prognosis is hampered due to resistance to chemotherapy and radiation. The median survival of GBM patients is around 15 months from diagnosis [1,2,3].

Temozolomide (TMZ) is an oral alkylating agent used as a first-line drug for chemotherapy of GBM [4]. TMZ is a pro-drug, and its cytotoxicity is derived from its metabolite 5-(3-methyltriazen-1-yl) imidazole-4-carboxamide (MTIC), which targets O6-meG, thereby leading to DNA mismatch repair (MMR) and subsequent DNA double-strand breaks (DSBs), cell cycle arrest and eventually apoptosis [5,6].

Multiple mechanisms have been identified in intrinsic or acquired (adapted) TMZ resistance [5]. Chemoresistance in GBM is largely attributed to the repair of TMZ-induced DNA lesions by O6-methylguanine-DNA methyltransferase (MGMT). The presence of MGMT enables cells to reverse the cytotoxicity of TMZ [7]. Initially, clinical research provided sufficient evidence that the lack of MGMT protein or epigenetic silencing by its promoter hypermethylation is correlated with longer survival [4]. Thus, the expression of MGMT is used as a parameter to predict the TMZ sensitivity of GBM patients. Recently, laboratory research proved that long-term treatment of TMZ induced MGMT expression and reduced susceptibility in a GBM cell line that naturally lacks MGMT, indicating the involvement of MGMT in acquired TMZ resistance [8]. In addition to MGMT, DNA repair mechanisms including MMR and DNA damage response (DDR) are also crucially involved in TMZ resistance. The MMR system is triggered upon TMZ treatment, thereby causing genomic instability [9]. Deficiency in MMR genes, for instance, due to mutation of *MSH2*, *MSH6* and *PMS2* [10] or inhibition of MMR [11], is associated with TMZ resistance [10,12], which may accelerate mutagenesis in resistant clones that could promote neoplastic progression [13]. The DDR has gained a great impact in cancer treatment and resistance [9]. TMZ-induced DNA damage triggers ataxia telangiectasia mutated (ATM) and Rad3-related (ATR) kinases in the DDR system, thereby affecting cell cycle checkpoints via p53-dependent and -independent (e.g., Chk1 and Chk2) pathways and eventually resulting in cell cycle arrest. Once the cycle has been arrested, a cell attempts to repair the damage [14]. This has encouraged the development of DDR inhibitors, e.g., ATM inhibitor (ATMi), as potential treatments. Collectively, expression of MGMT, impaired MMR, and activation of DDR signaling via ATM/ATR are all key factors causing TMZ resistance.

GBM is a heterogeneous tumor. A specific subset of glioma stem cells (GSCs) in the tumor is accountable for acquiring treatment resistance and turning it into a more aggressive form [15]. These GSCs possess self-renewal- and multipotent properties and are involved in tumor recurrence [16]. Moreover, TMZ treatment induces interconversion of a subset of GBM tumor cells to GSCs, which serves as an important mechanism of acquired TMZ resistance [17].

Acquired TMZ resistance is a severe limitation to more than 90% of recurrent gliomas showing no response to the second cycle of chemotherapy [18]. Until now, numerous studies have focused on TMZ resistance using established GBM cell lines that are naturally TMZ-sensitive versus TMZ-resistant upon genomic alterations in MGMT or p53, which enabled to unveil the mechanisms of inherited TMZ resistance [4,8,19]. However, regarding acquired TMZ resistance, there are only a few studies using acquired TMZ resistance models that usually take much time for cells to acquire resistance through chronic treatment with TMZ [8,20]. In the present study, we established an acute acquired TMZ resistance model in two GBM cell lines, U373 and LN229, that are naturally TMZ sensitive. The resistant cells derived from this model recapitulated all key features of TMZ resistance that could be maintained at least up to passage 15 of culture. Thus, this study established a promising acute acquired TMZ resistance model to study the mechanisms of TMZ resistance and to explore novel therapeutic drugs in the future.

## 2. Materials and Methods

### 2.1. Cell Culture, Generation of TMZ Resistant GBM Variants and Drug Treatment

LN229 and U373 cells were cultured in Dulbecco’s modified Eagle’s medium (DMEM) supplemented with 10% fetal bovine serum and 1 mM sodium pyruvate.

**Figure 1:**
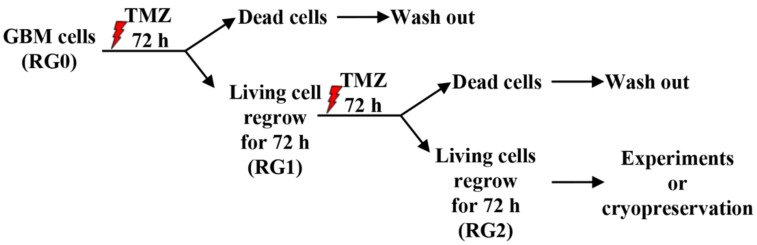
Schematic illustration of the acute acquired TMZ resistance model established in the present study. U373 and LN229 cells received 150 µM of TMZ treatment for 72 h (first cycle of treatment), and thereafter the damaged cells were washed out; viable cells regrew for 72 h and were named RG1. These RG1 cells underwent the second cycle of TMZ treatment for another 72 h. Then, the medium was removed, followed by washing out with fresh medium. The living cells were kept for regrowth for 72 h and were defined as RG2. All the RG variants generated in this study were used for different experiments until passage 15. Untreated parental cells in corresponding cell lines were defined as RG0 and used as control.

To generate TMZ resistant GBM cells, U373 and LN229 cells were treated with 150 µM of TMZ (Sigma, Steinheim, Germany, Cat# T2577) for 72 h as described as the most optimal condition in our previous study [21]. The damaged cells were removed by washout after the first cycle of treatment. The remaining viable and regrowth cells were cultured in fresh medium without (defined as RG1 cells) or with the second cycle of TMZ treatment (150 µM for another 72 h). After 72 h of the second cycle of TMZ treatment, damaged cells were washed out with fresh medium and the remaining viable cells were left to regrow for 72 h. These cells are defined as RG2 cells. RG variants generated in this study were used for different experiments until passage 15. Untreated parental cells in corresponding cell lines were defined as RG0 and used as controls. The two-cycle treatment model is schematically depicted in Figure 1.

### 2.2. Generation of GBM-RG Cells Expressing a 53BP1-Foci Reporter and Analysis of TMZ Induced Double-Strand Breaks (DSBs)

To visualize DSBs in single cells in response to TMZ treatment, a reporter expressing 53BP1 fused to mCherry fluorescent protein [22] was generated by stable transfection of 53BP1::mCherry plasmid (gift from Heidelberg University, Heidelberg, Germany) in U373- and LN229 cells.

GBM cells were seeded in 35 mm cell culture dishes at a confluence of 50% and incubated at 37 °C overnight. The transfection was performed by incubating cells with a mixture containing 1 µg of plasmid DNA and 2 µL of the Jet Prime DNA/siRNA transfection reagent (Polyplus-transfection, Illkirch, France) in 100 µL the jetPrime buffer for 8 h. Thereafter, the transfection medium was replaced with fresh growth medium supplemented with 5 µg/mL blasticidin for antibiotic selection. For monoclonal selection, 50 polyclonal cells were seeded in 96-well plate. Single clones were selected following microscopic evaluation. The selected monoclone was used to generate RG variants by two cycles of TMZ treatment as described above.

To quantify TMZ-induced 53BP1-foci in RGs, 53BP1::mCherry-GBM-RG cells were seeded on glass coverslip in 24-well plate and treated with TMZ or vehicle for 72 h. Treated cells were fixed in 4% paraformaldehyde followed by nuclear staining with DAPI. Cell images were acquired using an AxioImager M.2 microscope (Carl Zeiss AG, Oberkochen, Germany) with a 63× oil immersion objective. The number of 53BP1-foci per cell was counted in 100 individual cells per group. The number of total cells was determined in ten areas of interest to correlate to the number of cells with an accumulation of 53BP1-foci. Accumulation of 53BP1-foci was quantitatively analyzed using the ImageJ software (version 1.53q) [23].

### 2.3. MTT Assay

After 72 h of treatment with 150 µM TMZ or 10 µM ATMi (KU-60019) alone, or a combination treatment of TMZ and ATMi, or 0.2% DMSO as vehicle, the cell viability and the time course of cell regrowth were evaluated by MTT assay following the manufacturer’s instruction (Invitrogen, Darmstadt, Germany; cat# M6494). Briefly, cells were incubated with medium supplemented with 10% MTT for 3 h at 37 °C and 5% CO_2_. After dissolving formazan crystals in 50 µL of pure DMSO, the absorbance of formazan was measured using a microplate reader at 540 nm and at 630 nm as a reference. Relative cell growth was calculated by normalization of absorbance at each time point to a seeding control.

### 2.4. Flow Cytometry Detection of Cell Cycle and EdU Incorporation

To detect proliferating cells, an alternative thymidine analogue 5-ethynyl-2′-deoxyuridine (EdU) was used [24]. EdU has an alkyne group which enables covalent binding with fluorescent azide via copper-catalyzed reaction, namely, click chemistry. For EdU incorporation, cells were incubated in fresh growth medium supplemented with 15 µM EdU (Lumiprobe, Hannover, Germany; cat# 10540) for 1 h at 37 °C and 5% CO_2_. Cells were harvested, fixed in 70% ethanol or 4% paraformaldehyde, and permeabilized in 0.1% Triton X-100 in PBS. To label and detect incorporated EdU, cells were incubated with reaction cocktail containing 3 µM sulfo-Cy5-azide (Lumiprobe; cat# A3330), 2 mM CuSO_4_ (Sigma; cat# 209198) and 20 mg/mL ascorbic acid (Sigma; cat# A4544) in 100 mM Tris-PBS (pH 7.6) for 30 min at room temperature. After washing out the staining cocktail, cell nuclei were stained using 1.5 µg/mL DAPI in 0.1% Triton X-100 in PBS for 30 min at room temperature. For flow cytometry, CytoFLEX (Beckman Coulter) was employed with the setting as follows: 20,000 events for record and 10,000 events for display. DNA replication was analyzed using FlowJo based on histograms of EdU+/− cells. Cell cycle distributions by DNA contents were analyzed using FlowJo with the Dean-Jett Fox algorithm.

### 2.5. Apoptosis Assay by Nuclear Staining and Annexin V/PI Detection

For microscopic analysis, cells were stained with Hoechst-33258 and imaged in 4–6 randomized fields per well using an AxioImager M.2 microscope (Carl Zeiss AG, Oberkochen, Germany) with a 20× objective. Cells with condensed chromatin/apoptotic bodies were considered apoptotic.

For detection of TMZ-induced apoptotic cells by flow cytometry, cells (1 × 10^6^) were stained with FITC-Annexin V and Propidium Iodide (PI) using FITC Annexin V apoptosis detection kit I (BD Pharmingen, San Diego, CA, USA; cat# 556547) following the manufacturer’s protocol. For flow cytometry analysis (CytoFLEX), 30,000 events were recorded and 10,000 events were displayed. Early and late apoptotic cells are Annexin V-FITC+/PI− and Annexin V-FITC+/PI+, respectively.

### 2.6. Colony Formation Assay

RG cells (1 × 10^5^/well) were seeded in a 12-well plate and treated with TMZ alone, or concomitantly with 10 µM ATMi KU-60019 for 72 h. Treated cells were harvested and reseeded at a density of 200 cells/well and 500 cells/well for U373 and LN229 in a 12-well plate, followed by 11 days- and 7 days of incubation, respectively. Thereafter, cells were fixed with 4% paraformaldehyde and stained with 0.5% crystal violet. Stained colonies were digitally scanned, and their number and total area were analyzed using the ImageJ software (version 1.53q) [23].

### 2.7. Western Blot

Total protein extraction and western blot were performed as previously described [25]. The following primary antibodies were used: rabbit anti-cleaved caspase-3 (Cell signaling, Danvers, MA, USA; cat# 9664; 1:1000), rabbit anti-caspase 3 (Cell signaling; cat# 9662; 1:1000) (a gift kindly provided by Dr. Johann Matschke), and rabbit anti-GAPDH (Cell signaling; cat# 2118; 1:1000). After incubation with a secondary antibody (anti-rabbit IgG, HRP-linked; Cell signaling; 7074), the image on the blot was visualized by the ECL Select reagent (Amersham, Munich, Germany) and the image was acquired by using ImageQuant LAS 500 (GE Healthcare, Freiburg, Germany).

### 2.8. RT^2^-PCR

Total RNA was isolated using the innuPREP DNA/RNA Mini Kit (Analytic Jena AG). cDNA was synthesized using iScript cDNA Synthesis Kit (Bio-Rad, Munich, Germany). Real time-PCR was performed using 2× qPCRBIO SyGreen Mix with Fluorescein (PCRBIOSYSTEMS, London, UK) and the PCR program was set up as follows: initial denaturation at 95 °C for 2 min, 40 cycles of amplification at 95 °C for 5 s, and at annealing temperature for 25 s. Melting curve analysis was done using the following setting: 95 °C for 1 min, 55 °C for 1 min and 55–95 °C with a heating increase rate of 0.5 °C every 10 s. Primers and corresponding annealing temperatures used in the present study are listed in Table 1. Relative expression level was calculated by the 2^−ΔΔCT^ method and normalized to a reference gene, GAPDH or RPS13.

### 2.9. Neurosphere Assay

To test self-renewal of stem-like cells in serum free conditions, 100–200 cells/well were seeded in a 96 well suspension culture plate in quadruplicate for each treatment condition and incubated in DMEM; F12 (Gibco, Darmstadt, Germany) supplemented with 1× B27 minus Vitamin A (Gibco, Darmstadt, Germany), 20 ng/mL FGF-b and 20 ng/mL EGF (Peprotech, Cranbury, NJ, USA). Diameter- and the number of formed spheres were determined 11 d after seeding. For analysis, individual spheres bigger than 50 µm in diameter were counted as neutrospheres. Diameter was measured using the ImageJ software (version 1.53q) [23].

### 2.10. Statistics

Statistical analysis was performed using IBM SPSS Statistics 27 and GraphPad Prism 8. Data were presented as mean and standard deviation (mean ± SD). Differences between two and multiple groups were analyzed by Student’s *t*-test and by one-way ANOVA followed by the Scheffe test, respectively. A *p*-value < 0.05 was considered statistically significant.

## 3. Results

### 3.1. RG2 Cells Exhibit Resistance to Chemotherapy and Higher Regrowth Capacity in Post-Treatment Phase

**Figure 2:**
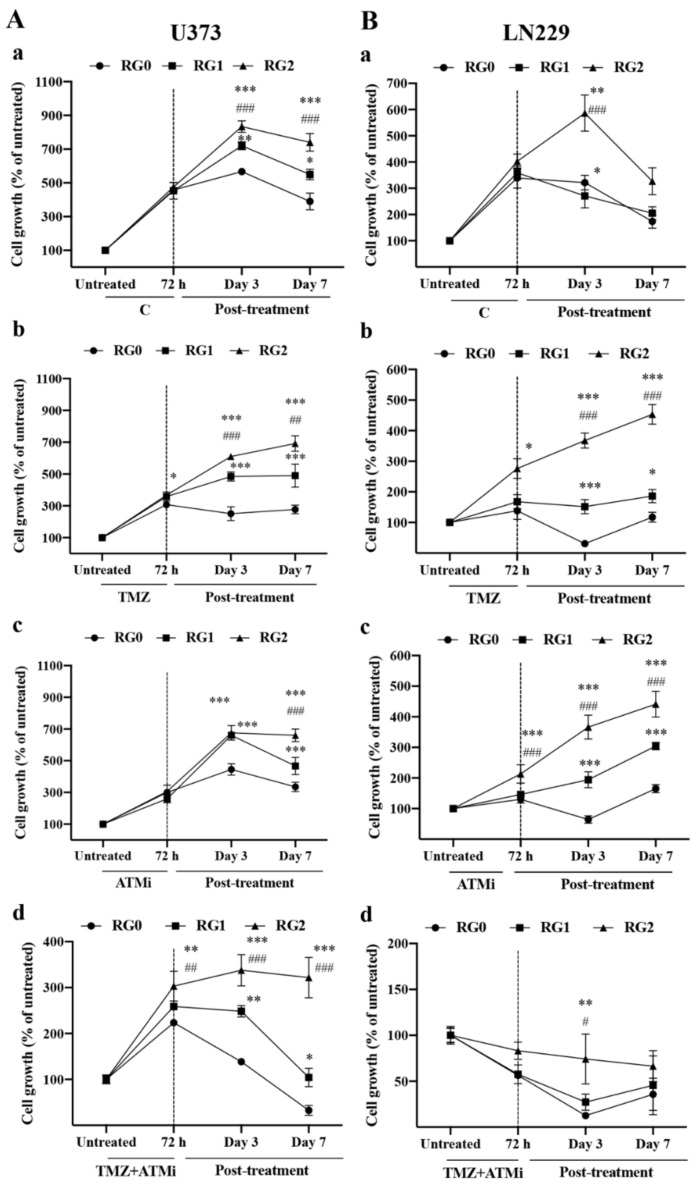
RG2 cells exhibit less susceptibility after TMZ treatment and higher regrowth capacity during the post-treatment period. U373 RG cells (**A**; passage 15) and LN229 RG cells (**B**; passage 12) received the treatment with 0.2% DMSO as vehicle control (C) (**A-a**,**B-a**), TMZ (150 µM) (**A-b**,**B-b**), ATMi alone (KU-60019, 10 µM) (**A-c**,**B-c**), or TMZ+ATMi (**A-d**,**B-d**) for 72 h, respectively. Thereafter, the media containing drugs were removed; viable cells were washed with fresh medium and cultured in normal growth medium for different periods (post-treatment). MTT assay was performed before (untreated, as seeding control), 72 h after treatment and at post-treatment time points as indicated. The results were reproduced in at least three independent experiments. RG0: regrown parental cells without TMZ treatment; RG1: regrown cells after one cycle of TMZ treatment; RG2: regrown cells after two cycles of TMZ treatment. *, *p* < 0.05; **, *p* < 0.01; ***, *p* < 0.001, compared with RG0; #, *p* < 0.05; ##, *p* < 0.01; ###, *p* < 0.001, compared with RG1.

To study whether RG cells resist TMZ cytotoxicity and sustain growth, U373-RG (Figure 2A) and LN229-RG (Figure 2B) cells were rechallenged with TMZ for 72 h, and thereafter the time course of cell regrowth was recorded after washing out the drugs. As shown in Figure 2, the growth rate of RG0, RG1, and RG2 after 72 h of treatment with vehicle (C) appeared similar among RG variants in both U373 (A-a) and LN229 (B-a) cells. However, RG2 (A-b,B-b) showed significantly higher viability after 72 h of TMZ treatment in comparison to the corresponding RG0 (*p* < 0.05) in both cell lines. These data indicate a significant resistance of RG2 cells to TMZ treatment, which was consistently observed in experiments performed with different passaged RG cells (Figure S1).

To observe the capacity of cell regrowth under drug free conditions, cell viability/proliferation assay was performed on day 3 and 7 of post-treatment after refreshing the medium. Interestingly, rapid cell regrowth was already detected on day 3. In U373 cells, TMZ treated RG2 cells regrew with an increase of 610% relative to the untreated control, which is significantly higher than its corresponding RG0 (251%) (*p* < 0.001) and RG1 (484%) cells (*p* < 0.001) (Figure 2A-b). A similar result was observed in LN229 cells. RG2 cells displayed a significant regrowth potency and the viability increased to 368% relative to the untreated control (*p* < 0.001, compared to RG0 and RG1), whereas RG0 and RG1 cells did not proliferate well (31% and 151% relative to untreated control, respectively) (Figure 2B-b). A sustained regrowth continued in the following 7 days in the post-treatment phase in RG0, RG1, and RG2 of both cell lines. Among the RGs in U373, RG2 exclusively showed the most potent and highest regrowth rate (*p* < 0.001 and *p* < 0.01, vs. RG0 and RG1 on day 7, respectively) (Figure 2A-b). Similar data were obtained in LN229 cells (Figure 2B-b). Of note, a decrease in cell growth was observed in the vehicle treated RGs of both cell lines on post-treatment day 7, which was due to overconfluent cells resulted from long term of culture period. (Figure 2A-a,B-a).

The impacts of acquired TMZ resistance on cell viability and regrowth in response to combination chemotherapy using TMZ and ATMi (KU-60019) were also studied. Inhibiting the ATM pathway with concomitant treatment of TMZ may act synergistically in a prolonged manner even during post-treatment, thereby serving as a more severe treatment strategy to kill or suppress the proliferation of e.g., cancer progenitor cells that are more resistant to chemotherapy. To this end, U373-RG and LN229-RG cells were treated with ATMi alone or together with TMZ for 72 h. Interestingly, when treated with ATMi alone, RG2 cells in both cell lines regrew more rapidly compared to RG0 cells (Figure 2A-c and Figure 2B-c for U373- and LN229, respectively). For the combination treatment, U373-RG2 cells showed a significantly higher growth rate than RG1 and RG0 72 h after the combination treatment (Figure 2A-d; *p* < 0.01). The regrowth potency of U373-RG2 cells was even more significantly detected on day 3 (*p* < 0.001, vs. RG0 and RG1) and day 7 (*p* < 0.001, vs. RG0 and RG1) in the post-treatment phase. Obviously, LN229 cells were more sensitive to TMZ+ATMi treatment, but LN229-RG2 cells still showed a higher regrowth capacity on day 3 of post-treatment compared to RG0 (*p* < 0.01) and RG1 (*p* < 0.05) (Figure 2B-d).

Taken together, as a proof of principle study, the MTT assay demonstrated that U373 and LN229 cells acquired acute TMZ resistance after two cycles of TMZ challenge. TMZ resistant RG2 cells were less susceptible not only to TMZ treatment but also to ATMi and to the combination treatment of TMZ+ATMi than parental cells; RG2 cells also regrew faster than parental cells during the post-treatment phase.

### 3.2. RG2 Cells Exhibit Enhanced Clonogenic Growth Capacity Accompanied by Higher Proliferation Activity in the Post-Treatment Phase

**Figure 3:**
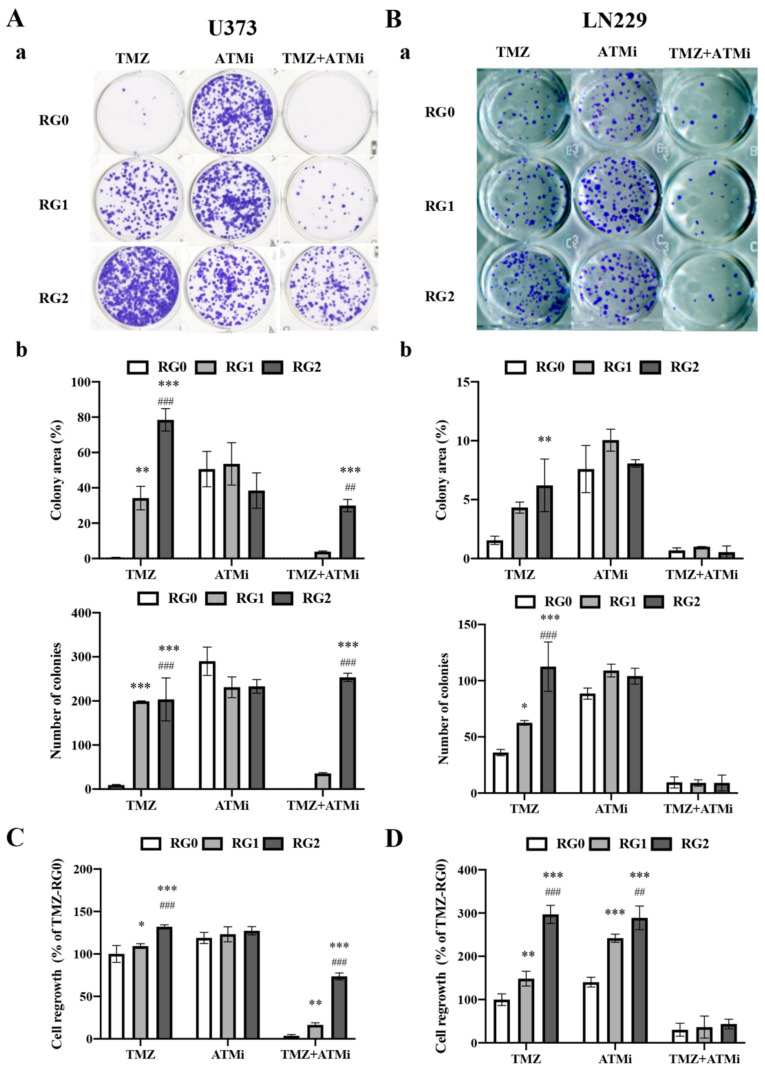
RG2 cells display a remarkable clonogenic growth accompanied by higher proliferation activity in the post-treatment phase. U373 RG cells and LN229 RG cells received treatment with TMZ (150 µM), ATMi (KU-60019, 10 µM), or TMZ+ATMi for 72 h. After the treatment, viable cells were reseeded in a 12-well plate for colony formation assay or in a 96-well plate for proliferation assay. (**A**,**B**) Colony formation assay. After incubation of reseeded U373-RG cells (**A**) and LN229 RG cells (**B**) for 11 d and 7 d, respectively, cells were stained with 0.5% crystal violet for analysis of the number- and the total area of colonies using the ImageJ software (version 1.53q). (**A-a**,**B-a**): Representative images showing colonies grown in different groups of U373 RGs and LN229-RGs, respectively. (**A-b**,**B-b**): Quantitative analysis of the area and the number of colonies in U373 RGs and LN229-RGs, respectively. (**C**,**D**) Regrowth of RG cells. MTT assay was performed on day 11 and day 7 of post-treatment in TMZ-treated U373-RGs and LN229 RGs, respectively. *, *p* < 0.05; **, *p* < 0.01; ***, *p* < 0.001, compared with RG0. ##, *p* < 0.01; ###, *p* < 0.001, compared with RG1.

Clonogenic assay is a method to test the ability of single cells in a population to undergo “unlimited” division. Thus, it has been widely used to determine cell death after treatment with cytotoxic agents. Here, we studied the clonogenicity of RGs after chemotherapy. Cells received TMZ, ATMi, or combination treatment of TMZ and ATMi for 72 h. After drugs were washed out, treated cells were reseeded in fresh media for colony formation assay and proliferation assay. Figure 3 represents colony formation in U373-RGs (A) and in LN229-RGs (B) on day 11 and day 7 of post-treatment with TMZ or ATMi or with TMZ+ATMi, respectively. Quantitative analysis of the area and the number of colonies revealed a significantly more colony formation in U373-RG2 cells than in RG1 and RG0 cells under both treatment conditions with TMZ alone and TMZ+ATMi (Figure 3A-b) (*p* < 0.001). Increased colony formation in terms of both number (*p* < 0.001) and area (*p* < 0.01) was also seen in LN229-RG2 and RG1 cells compared to RG0 (Figure 3B-b). Due to the sensitive response of LN229 cells to severe damage caused by the combination treatment, the colony formation ability of all LN229 RGs was largely restricted, which is representative of poor clonogenicity. Nevertheless, RG2 cells showed a trend of forming more colonies than RG0 and RG1 despite no statistical significance given (Figure 3B-b). However, none of the RGs in both cell lines showed any significant difference in clonogenic growth upon treatment with ATMi alone (*p* > 0.05).

In parallel to the colony formation assay, cell proliferation was investigated in the same experimental setting. U373-RG2 cells exhibited a significantly higher proliferation activity in comparison to RG0- and RG1 cells (both *p* < 0.001) in the groups of TMZ alone or TMZ+ATMi, but not in the group of ATMi alone on post-treatment day 7 (Figure 3C). In LN229-RGs, similar results were found after TMZ treatment. However, LN229-RG2 and -RG1 cells treated with ATMi alone showed a significantly higher growth capacity compared to RG0. Moreover, no LN229-RG cells grew well after combination treatment with TMZ and ATMi (Figure 3D).

Taken together, a larger population of RG2 cells in both cell lines displayed TMZ resistant features to grow into colonies and sustainably undergo proliferation.

### 3.3. RG2 Cells Show Less DNA Damage in Individual Cells after TMZ Treatment in Association with the Downregulation of MMR Gene Expression

**Figure 4:**
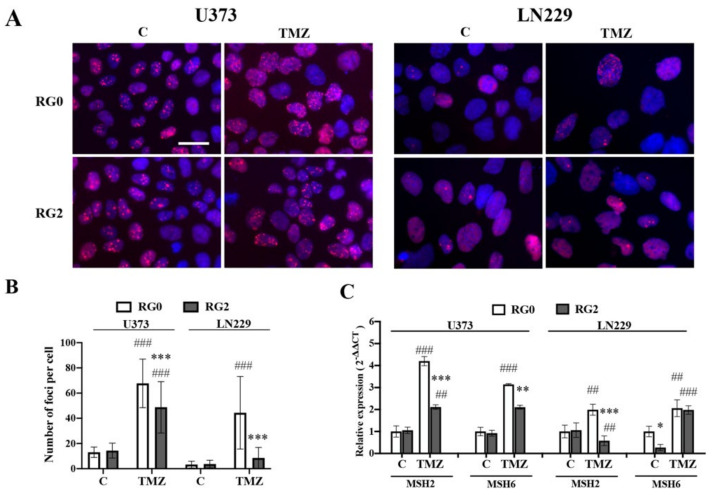
RG2 cells show less DNA damage in individual cells after challenge with TMZ, which was associated with the downregulation of MMR gene expression. 53BP1::mCherry-transfected U373-RGs and -LN229-RGs were treated with TMZ (150 µM) or vehicle. DNA double-strand breaks (DSBs) and the expression of MMR genes were evaluated 72 h after treatment. (**A**) Representative images show the accumulation of DSBs as evidenced by the 53BP1-foci in U373-RGs and LN229-RGs. Scale bar: 30 μm. (**B**) Quantitative analysis of accumulated 53BP1-foci. The number of 53BP1-foci was counted in 100 individual cells per group. (**C**) Downregulation of *MSH2* and *MSH6* genes in RG2 cells detected by RT^2^-PCR. *, *p* < 0.05; **, *p* < 0.01; ***, *p* < 0.001, compared with RG0. ##, *p* < 0.01; ###, *p* < 0.001, compared with a corresponding vehicle control C.

TP53 binding protein 1 (53BP1) serves as a marker for the number/location of DSBs recruited by the ATM/ATR pathway [26]. To explore whether DNA mismatch repair upon TMZ treatment is influenced by the acquired TMZ resistant nature of RG2, we generated 53BP1 reporter cell lines, 53BP1::mCherry-U373 and -LN229, that express recombinant 53BP1 fused to mCherry under the control of the EF1α promoter. Following our standard protocol, RG variants expressing the 53BP1 reporter were generated and exposed to TMZ for 72 h to quantify DSBs. Figure 4A shows 53BP1-foci accumulated in cell nuclei in response to TMZ, which is more pronounced in RG0 cells than in RG2 in both U373 and LN229 cells. Further quantitative analysis confirmed a significant increase in the number of 53BP1-foci in TMZ-treated RG0, but less in RG2 of both cell lines (*p* < 0.001). Upon vehicle treated (C), there was no significant difference in the number of 53BP1-foci between RG0 and RG2 (Figure 4B).

Next, we determined the expression of MMR genes—*MSH2* and *MSH6*—in RG0 and RG2 cells after 72 h of TMZ treatment. As shown in Figure 4C, TMZ treatment resulted in 4.2- and 3.1-fold upregulation of *MSH2* and *MSH6* expression, respectively, in U373-RG0 compared to the control (C). These two genes were upregulated to a much lesser extent in U373-RG2 (2.1 and 2.1 fold for *MSH2* and *MSH6*, respectively) than in RG0 (*p* < 0.001 and *p* < 0.01, respectively). Similarly, while expression of *MSH2* and *MSH6* increased in LN229-RG0 cells in response to TMZ treatment, a lower expression of *MSH2* (*p* < 0.05) but not *MSH6* was detected in LN229–RG2 cells. In addition, the impact of ATMi treatment on the expression of MMR genes was also determined. ATMi treatment resulted in a significant upregulation of *MSH2* and *MSH6* expression in both U373-RGs and LN229-RGs compared to the control (C). This upregulation was similarly seen in RG0 and RG2 of both types of cells excepting a higher expression of *MSH2* in LN229-RG0 compared to -RG2 (Figure S2).

### 3.4. RG2 Cells Show a Reduction in Cell Cycle Arrest at G2/M Accompanied by an Increase in DNA Replication after TMZ Treatment

Characterization of chemoresistant features, including susceptibility, sustained regrowth, and clonogenicity in RG2 and RG1, highlights the potential of RG2 as the most TMZ resistant model in this study. Thus, we focused on RG2 in the subsequent studies.

**Figure 5:**
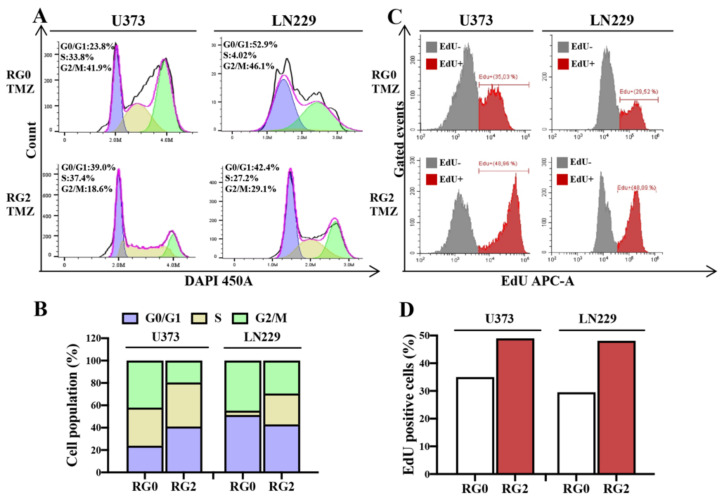
RG2 cells present less G2/M arrest accompanied by an increase in DNA replication after TMZ treatment. Cell cycle (**A**,**B**) and DNA replication (**C**,**D**) were detected in U373-RGs and LN229-RGs cells 72 h after TMZ (150 µM) treatment by flow cytometry. (**A**) Cell cycle histogram. After staining with DAPI, cells underwent flow cytometry assay. Each cell cycle phase is shown in the histogram of DNA contents: G0/1- (purple), S- (yellow) and G2/M phase (green) modeled with the Dean-Jett Fox algorithm. (**B**) Stacked bar graph of cell cycle distributions seen in (**A**). RG2 showed a redistribution in cell cycle compared with RG0. A significantly reduced cell population at the G2/M arrest phase was observed in RG2 of both cell lines after treatment with TMZ. (**C**) Histogram of DNA replication detected by flow cytometry. Two populations of cells—EdU negative (EdU−, grey) and positive (EdU+, red)—were detected after EdU incorporation. (**D**) Bar graph of EdU+/− populations seen in (**C**). EdU population analysis revealed an increase in DNA replication (EdU+) in RG2 cells compared with RG0 in both cell lines after treatment with TMZ. The data are representative of three independent experiments.

TMZ leads to DNA mispairs during DNA replication and consequently cell cycle arrest in the G2/M phase and apoptosis [27]. To address whether acquired TMZ resistance attenuates TMZ-induced cell cycle arrest and eventually prevents apoptosis, DNA contents-based cell cycle assay was performed following TMZ treatment. In vehicle controls, RG2 and RG0 showed similar cell cycle distributions in both U373 and LN229. However, TMZ-treated U373-RG2 showed a remarkable decrease in the fraction of G2/M phase (18.6%) in comparison to its RG0 counterpart (41.9%) (Figure 5A). A similar redistribution of cell cycles was observed in LN229 cells. The percentage of cells at G2/M decreased from 46.1% to 29.1% upon TMZ treatment in this cell line (Figure 5A). Flow cytometry revealed a clear redistribution of the cell cycle when cells acquired TMZ resistance, and the most significant alteration in G2/M arrest phase was defined in RG2 of both cell lines (Figure 5B). It is noteworthy that cell cycle redistribution in RG2 was accompanied by a reduction in TMZ-induced cell death (Figure S3).

RG2 cells grew rapidly, even when exposed to TMZ (Figure 2), and appeared to evade cell cycle arrest (Figure 5A,B). To understand the effects of TMZ resistance on DNA replication in response to TMZ treatment, EdU incorporation was performed, followed by flow cytometry. In both cell lines, RG2 showed a greater fraction of EdU positive (EdU+) cells than the corresponding RG0 (Figure 5C). The EdU+ fraction was 35.03% and 48.95% in U373-RG0 and U373-RG2, respectively. LN229-RG2 showed 48.09% of EdU+ populations, which is more than in LN229-RG0 cells (29.52%) (Figure 5D). These findings suggest that more RG2 cells escaped from G2/M arrest and underwent proliferation than RG0 despite TMZ treatment. This is also in line with the findings of the MTT (Figure 2) and colony formation (Figure 3) assays.

### 3.5. Apoptosis Is Involved in the Chemoresistance of U373-RG2 and LN229-RG2 Cells via Different Pathways

**Figure 6:**
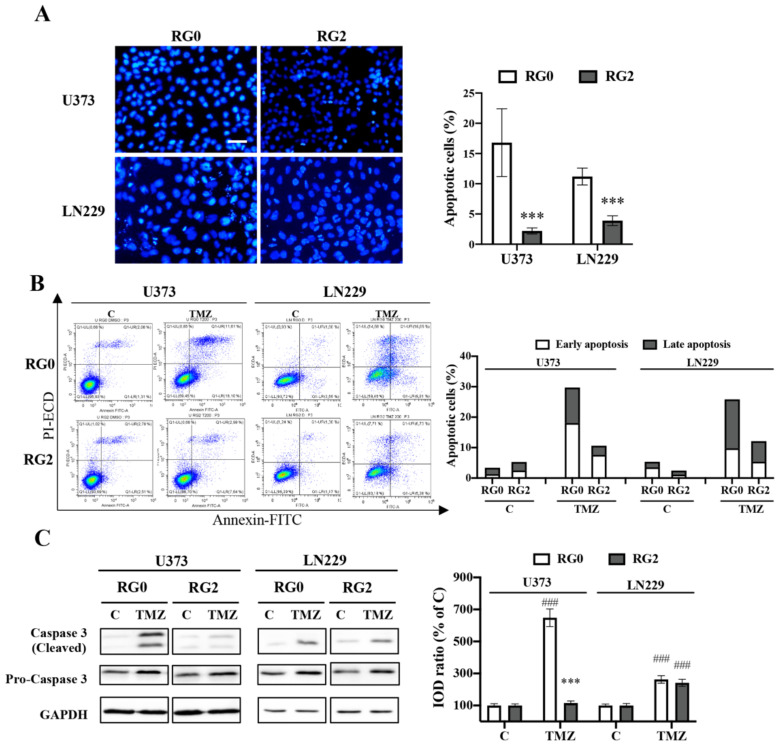
RG2 cells evaded TMZ-induced apoptosis. Apoptosis was evaluated in U373-RGs and LN229-RGs after 72 h of TMZ (150 µM) treatment. (**A**) Apoptosis was detected by nuclear staining with Hoechst 33258. Cells exhibiting condensed chromatin or apoptotic bodies were counted as apoptotic cells. Quantitative analysis was performed in six random fields per well per group (in quadruplicate). Scale bar: 50 μm. ***, *p* < 0.001, compared with RG0. (**B**) Apoptosis assay by staining with Annexin V/PI followed by flow cytometry. Dot plots show apoptotic populations in RGs of U373 and LN229 cells (left) after 72 h of treatment with TMZ (150 µM) or vehicle. Bar graph shows the percentage of early and late apoptotic cells in total cells from the dot plots (right). (**C**) TMZ-induced caspase 3 activation was detected by western blot (left) and semi-quantitation (right). The results were reproduced in at least three independent experiments. ***, *p* < 0.001, compared with RG0. ###, *p* < 0.001, compared with a corresponding vehicle control (**C**).

Given that TMZ-treated RG2 exhibited a reduction in the subG1 phase relative to the corresponding RG0 (Figure S3), we next investigated whether TMZ-induced apoptosis was hampered in RG2 cells. To this end, following 72 h of TMZ treatment, apoptotic cells were determined by nuclear staining, followed by quantification of condensed chromatin and apoptotic bodies and by Annexin V/PI staining followed by flow cytometry. Nuclear staining demonstrated significantly fewer apoptotic cells in RG2 than in RG0 in response to TMZ treatment (*p* < 0.001) in both cell lines (Figure 6A). Quantification of apoptotic nuclei revealed 16.8% and 2.2% of apoptotic cells in U373-RG0 and U373-RG2 cells, respectively. Similar results were found in LN229 cells. In line with this finding, flow cytometry-based assay also showed a smaller apoptotic cell population in U373-RG2 (10.63%) compared to U373-RG0 (29.71%). In LN229 cells, the fraction of apoptotic cells was 25.86% and 12.11% in RG0 and RG2, respectively (Figure 6B).

To test the molecular feature in TMZ induced apoptosis in RGs, the active form of caspase 3 (cleaved caspase 3) and the pro-caspase-3 were detected by immunoblotting (Figure 6C, left panel; Figure S5). A pronounced activation of caspase 3 was detected 72 h after TMZ treatment, which was attenuated in U373-RG2 cells but not in LN229-RG2 cells. The expression of pro-caspase 3 did not differ among tested groups. Semi-quantification of the blots using GAPDH as reference indicated a six-fold decrease in caspase 3 activation in U373-RG2 compared to U373-RG0 (Figure 6C, right panel). However, suppression of caspase 3 activation was not detected in LN229-RG2 cells, suggesting caspase 3 activated by TMZ treatment in LN229-RG2 might not be involved in its TMZ resistant effect. Semiquantification using pro-caspase 3 as reference showed similar results.

### 3.6. U373 RG2 Cells Exhibit Enhanced Self-Renewal Capacity in Association with Upregulation of Stem Cell Markers

**Figure 7:**
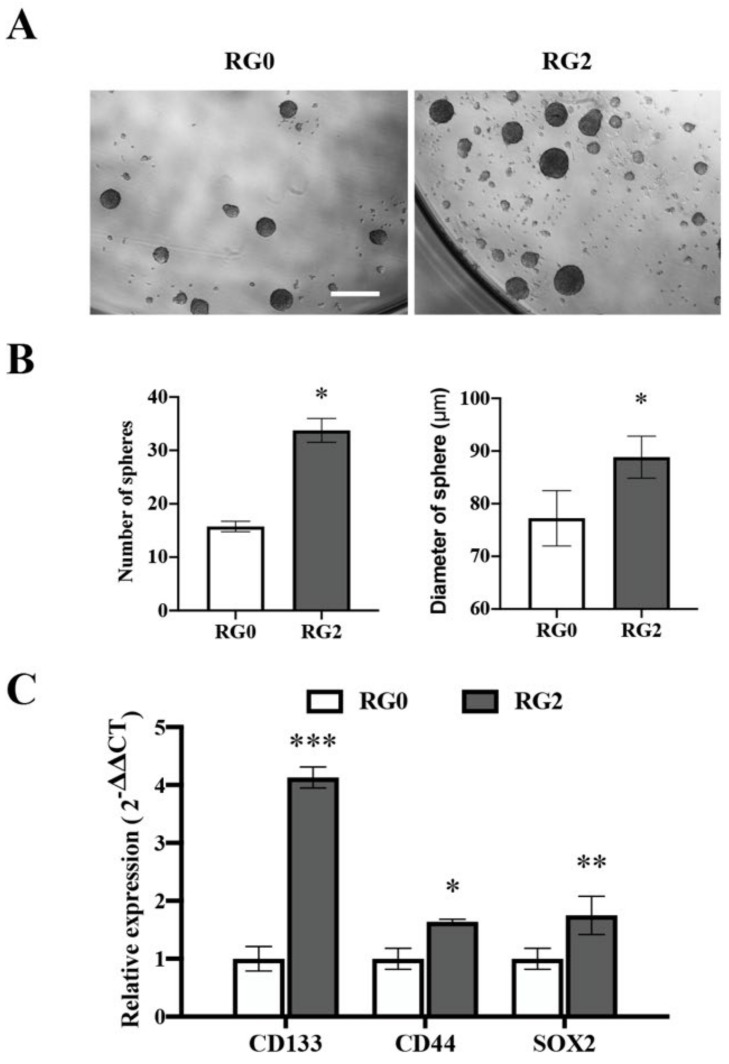
U373 RG2 cells exhibit enhanced self-renewal associated with an upregulation of stem cell markers. Neurosphere assay was performed in U373-RG cells. (**A**) Representative images of neurospheres derived from U373-RG0 and -RG2 cells. Scale bar: 200 µm. (**B**) Quantitative analysis of neurospheres. RG2 cells formed more (left) and bigger (right) neurospheres compared with RG0 cells. (**C**) Expression of stem cell markers were detected by RT^2^-PCR. RG2 cells expressed significantly higher levels of *CD133*, *CD44* and *SOX2* in comparison with RG0. *, *p* < 0.05; **, *p* < 0.01; ***, *p* < 0.001, compared with RG0.

Next, we investigated whether RG2 cells possess cancer stem cell (CSC) properties. To this end, neurosphere assays were performed in U373-RGs under serum free conditions. Figure 7A shows the representative images of spheres derived from U373-RG0 and -RG2 cells. Quantitative analysis revealed around twice as many spheres formed in RG2 as in RG0 (Figure 7B; left panel; *p* < 0.05). RG2-derived spheres also appeared larger than those derived from RG0 (Figure 7B; right panel; *p* < 0.05). These findings are in line with the observation of the augmented clonogenicity of RG2 (Figure 3). To further validate the stem-like phenotype of RG2 cells, we attempted to determine whether the gene expression signature was altered in RG2 cells to evoke stem-like behavior. RT^2^-PCR detected a significant upregulation of *CD133* (*p* < 0.001), *CD44* (*p* < 0.05), and *SOX2* (*p* < 0.01) in U373-RG2 compared with -RG0 (Figure 7C). In LN229-RGs, the expression of *CD44* and *SOX2* was very low and CD133 was even undetectable. Moreover, there was no significant difference in the expression of these genes between LN229-RG2- and -RG0 cells (Figure S4). This aspect is consistent with the observation that LN229 RG cells hardly formed spheres.

## 4. Discussion

**Figure 8:**
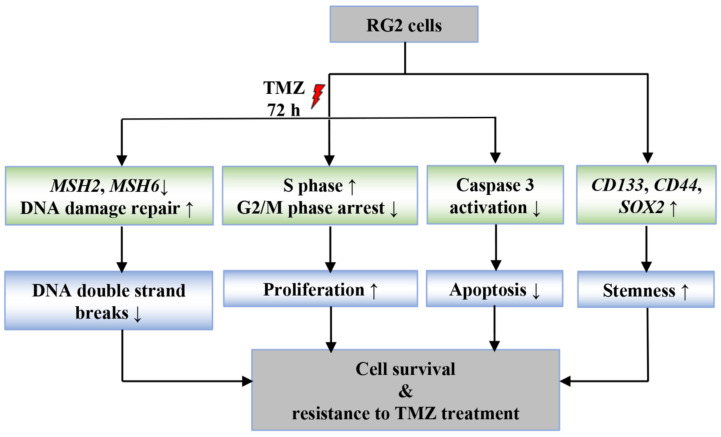
Characterization of the phenotype and related gene signatures in TMZ resistant GBM cells. Upon treatment with TMZ, RG2 cells showed less susceptibility and sustained growth, which resulted from reduced DSBs and downregulation of *MSH2* and *MSH6*, evasion from G2/M arrest and increased DNA replication (proliferation) and decreased apoptosis. Moreover, these TMZ resistant cells presented stem cell properties, as evidenced by rapid clonogenic regrowth, higher cell self-renewal capacity and upregulation of multiple stemness-related genes. All these findings highlight that our TMZ resistance model represents the key features and underlying mechanism of acquired TMZ resistance.

TMZ is the first-line chemotherapeutic drug in the standard care for GBM. However, relapse occurs due to inherited and/or acquired resistance to TMZ. Inherited TMZ resistance is closely associated with the status of MGMT or p53 in cancer cells [4,28]. In recent decades, there have been efforts to unravel molecular events in acquired TMZ resistance in various models. Previous acquired TMZ resistance models were usually generated by exposing GBM cell lines or primary tumor cells to TMZ continuously or in a stepwise manner with gradually increasing concentrations for up to several months [4,8,19]. In the present study, we established an acute model of acquired TMZ resistance in GBM cell lines, by which TMZ resistant phenotypes and underlying mechanisms were investigated (Figure 8). We found that in comparison to the parental cells (RG0), RG2 cells appeared more resistant to chemotherapy (TMZ or TMZ+ATMi). Moreover, challenged RG2 cells regrew rapidly and sustainably under drug free conditions during the post-treatment period. These features bear the properties of acute acquired TMZ resistance in RG2 cells and suggest two-cycle treatment with TMZ as a valuable model to induce TMZ resistance. Further studies revealed the mechanism involved in TMZ resistance of RG2 cells. In response to TMZ treatment, RG2 cells displayed high clonogenic regrowth- and proliferation capacity, a reduction in G2/M arrest, an increase in DNA replication, attenuated DNA damage linked to downregulation of MMR genes *MSH2* and *MSH6*, and subsequently reduced apoptosis via inhibition of caspase 3 activation. In addition, RG2 cells upregulated the expression of stem cell markers such as CD133, CD44, and SOX2 and formed more and bigger spheres, denoting stem cell-like properties of RG2 cells derived from our acute acquired TMZ resistance model (Figure 8).

Sustained cell proliferation is one of the characteristics involved in TMZ resistance and tumor recurrence. In comparison to the parental cells (RG0), TMZ resistant RG2 cells showed less susceptibility to challenges with TMZ or with TMZ + ATMi. More importantly, RG2 cells rapidly regrew after treatment, suggesting tumor initiating/propagating potential of RG2 cells (Figure 2). This hypothesis was firmly validated by a colony formation study (Figure 3). The findings demonstrate that RG2 cells acquired resistance against the TMZ cytotoxic effect of a new round of chemotherapy.

It is known that TMZ causes DNA damage and subsequent activation of the DNA damage response kinases (DDR) (i.e., ATM, ATR, and DNA-PK). Aberrant activation of these DDR proteins in cancer is strongly correlated with resistance to genotoxic anti-tumor therapeutics in cancer cells [29]. Indeed, an association of ATM with the sensitivity of glioma cells to TMZ has been reported [30]. Therefore, adding a DDR inhibitor to standard therapy may serve as an effective combination therapy for GBM. In the present study, we also explored the response of RG0, RG1, and RG2 cells to the combinational treatment of TMZ with ATMi (KU-60019). As predicted, the concomitant treatment of TMZ and ATMi led to synergistic cytotoxicity (Figure 2A-d,B-d) and attenuated clonogenic survival and growth (Figure 3) in all RGs. Despite a more severe damage effect of the combination treatment, the viability of U373-RG2 cells at 72 h of treatment was significantly higher (*p* < 0.01, Figure 2A-d) than with TMZ treatment alone (*p* < 0.05, Figure 2A-b), indicating a stronger chemoresistance of RG2. A different response to TMZ+ATMi treatment was observed post-treatment under drug free conditions among U373-RG0, -RG1, and -RG2 cells (Figure 2A-d) and between U373 (Figure 2A-d) and LN229 (Figure 2B-d) cells. Strong regrowth potency was detected in U373-RG2 throughout the post-treatment period (from day 3 to 7) but not in U373-RG1 and -RG0 cells. This phenomenon may imply a more activated intrinsic resistance mechanism in RG2 cells in response to this combination treatment. In fact, all LN229 cells stopped proliferating after treatment with TMZ+ATMi and did not appear to have regrowth potential in the post-treatment phase (Figure 2B-d). Thus, the synergistic cytotoxicity of this combination treatment affected the viability of LN229-RGs more dramatically than in U373-RGs cells. These findings are agreement with the notion of cellular lethality of ATM inhibition in a p53-dependent manner. The effect of ATM depends on the state of p53. U373 is naturally p53 mutated, whereas LN229 expresses normal functional p53 [31,32,33]. Nevertheless, a faster regrowth was still observed in LN229-RG2 cells on day 3 post-treatment (Figure 2B-d). Taken together, these data suggest that the acquired resistant RG2 cells were less susceptible not only to TMZ but also to the combinational treatment. Although ATMi is in clinical trial for brain cancers only (available online: https://clinicaltrials.gov/ct2/show/NCT03423628; accessed on 10 January 2022), our findings may raise caution as to the consideration of whether adding ATMi in chemotherapy together with TMZ is beneficial to those GBM patients who had failed from TMZ treatment. In addition, given the p53-dependent effect of ATM, the necessity of personalized therapy should be emphasized when ATMi is added to standard chemotherapy in the future.

After characterization of the acute acquired TMZ resistance in RG2 cells, we focused on the mechanisms involved in the resistance and regrowth of RG2 cells. TMZ is an alkylating agent that is converted to its active form which then targets O6-meG. O6-meG is crucial for MMR and cell survival. The MMR system prevents base substitutions and repeated genomic instability [9]. MSH2 and MSH6 are MMR proteins and can recognize mismatches resulting from TMZ induced methyl adduct. Cells with defective MMR are more resistant to alkylating agents [34,35,36]. Thus, we next checked the genome integrity and the MMR in acquired TMZ resistant RG2 cells. Compared to corresponding vehicle control groups, TMZ treatment resulted in accumulation of DSBs in nuclei (Figure 4B) accompanied by an upregulation of *MSH2* and *MSH6* (Figure 4C) in both RG0 cells and resistant-RG2 cells. This is inconsistent with a previous report, in which enrichment of MMR genes was detected in patient-derived glioblastoma stem cells (GSCs) treated with high doses of TMZ [37]. Meanwhile, we noted significantly fewer DSBs and less increase in *MSH2* and *MSH6* expression at 72 h after TMZ treatment in RG2 cells expressing the 53BP1 reporter compared to corresponding parental RG0 cells (Figure 4A,B, *p* < 0.001 for both cell lines). Therefore, noticeable downregulation of those MMR genes in RG2 cells may lead to fewer TMZ-induced DSBs, thereby resisting chemotherapy.

LN229 [20,38] and U373 [39,40] cells have a high percentage of MGMT promoter methylation and are therefore MGMT deficient. Melguizo et al. [20] reported a slight change in promoter methylation of MGMT (100% to 75%) in LN229 cells after TMZ treatment. However, this event was not enough to express MGMT and at least not at a detectable level by western blot. In line with these data, we assume that our RG cells unlikely express MGMT.

TMZ preferentially induces cell cycle arrest in the G2/M phase [41,42], involving both ATM and MMR systems [5,6,43]. However, such molecular events were suggested to rely on a different model [30]. In our model, RG2 cells exhibited less G2/M arrest (Figure 5A) accompanied by higher DNA replication (Figure 5B) after 72 h of TMZ treatment in comparison to the corresponding parental cells (RG0), which proves the resistant property of RG2 cells. Interestingly, cell cycle arrest at G2/M was similarly observed in RG2 derived from p53-deficient U373 and p53-proficient LN229, suggesting that TMZ administration spurred G2/M arrest, more relying on the ATM-Chk2-CDC25A-CDk1 pathway [44]. This is important when considering the combination of TMZ with inhibitors of enzymes involved in S, G2/M, or M phase checkpoints to increase sensitivity to chemotherapy. Accumulating evidence supports cell cycle checkpoint kinases as valuable targets for GBM therapy. Our resistant model could be helpful for future studies concerning the inhibition of kinases involved in the S and G2/M checkpoints to increase mitotic failure.

In many cases, cell cycle arrest gives time to repair cell damage (e.g., DNA damage, oxidative stress). If repair mechanisms fail, cells undergo apoptosis [45]. Considering a significant downregulation of *MSH2* and *MSH6* in RG2 compared to RG0, we supposed that shortened cell cycle arrest was associated with less apoptosis in RG2 cells. As expected, apoptosis upon TMZ treatment was significantly detected in RG0 cells but to a much lesser extent than in RG2 in both cell lines (Figure 6A,B), indicating a potent resistance of RG2 cells to apoptosis induced by TMZ. Interestingly, a dramatic inhibition of caspase 3 activation was found in U373-RG2 but not in LN229 cells (Figure 6C), suggesting a caspase 3 dependent resistance to apoptosis in U373-RG2, whereas TMZ induced apoptosis in LN229 cells is more likely regulated via a caspase 3-independent mechanism. This finding is consistent with the different status of p53 in these two cell lines: mutated p53 in U373 cells vs. functional p53 in LN229 cells. TMZ induces apoptosis via O^6^-meG in a p53-dependent manner [46]. p53-mutated GBM cells are less sensitive to TMZ than wild-type cells through the Bcl-2/caspase pathway, whereas p53 wild-type cells trigger the death receptor pathway by induction of the Fas receptor [46]. Thus, our TMZ resistant model in U373 and LN229 cells accordingly differentiated the underlying mechanism of apoptosis induced by TMZ, emphasizing the influence of the genomic background of cells on the study of inhibition of apoptosis using caspase 3 inhibitors.

GSCs are involved in GBM initiation, progression, and therapeutic relapse [47,48,49]. A small quiescent or slow cycling subpopulation of non-GSC in vitro or in tumor could interconvert to GSCs, which is accelerated by the alteration of the microenvironment (e.g., hypoxia) [50] and by long-term TMZ exposure. These newly formed GSCs acquire phenotypic and functional characteristics similar to those of native GSCs. The TMZ-mediated expansion of the GSC pool is driven by newly converted GSCs that express molecular markers associated with the parental GSCs [17]. GSC populations are more chemoresistant than their non-GSC counterparts [51]. However, there are also diverse findings from in vitro models recapitulating TMZ resistance. For example, a transient model of TMZ resistance in U251 did not show any alteration in expression of EMT genes, including *CD44* and stemness genes including *SOX2*, compared with parental cells [8]. In the present study, TMZ resistant RG2 cells derived from the acquired resistance model also showed comparable features as to those of known GSCs (Figure 7A). Compared with parental cells (RG0), RG2 cells possessed a better capacity of sphere formation and growth under serum free conditions (Figure 7B). These RG2 cells expressed significantly higher levels of stem cell markers, including *CD133*, *CD44*, and *SOX2*, compared to RG0 cells (Figure 7C). The upregulation of these stem markers is correlated with less susceptibility to TMZ via diverse signaling pathways [20,52,53,54]. Based on the alteration in their stem-like gene expression signature together with the GSCs-like neurosphere forming capacity and clonogenic growth, RG2 cells may serve as an important tool to understand mechanisms underlying the chemoresistance linked to their potency as tumor initiating/propagating cells.

## 5. Conclusions

TMZ has become a cornerstone of GBM treatment. Despite the highly heterogeneous and mutation-prone nature of GBM, TMZ resistance is quite common for this lethal tumor, and it is, unfortunately, a key factor in tumor resistance and recurrence. This resistance can be either inherently characteristic of certain tumors or acquired after initial treatment. The present study established an acute acquired TMZ resistance model, which recapitulated all key features of TMZ resistance, including less susceptibility and sustained regrowth, increased clonal growth and DNA replication, reduced DSBs and impaired MMR, reduced G2/M arrest, and evasion of apoptosis after a new round of TMZ treatment. Moreover, the TMZ resistant cells derived from our model presented the stem cell property that is crucial for their TMZ resistance. Taken together, the present study introduces a valuable model for the future study of mechanisms underlying TMZ resistance and for exploration of new therapeutic drugs or better treatment strategies.

## Figures and Tables

**Table 1:** Primer sequences and annealing temperatures for real-time reverse-transcription PCR (RT^2^-PCR).

Primer Name	Sequence	Annealing Temperature (°C)
MSH2		60
for.	TTTACCCGGAGGAGAGACTGC	
rev.	TGCTCTCCCTTTTTGCCTTTC	
MSH6		60
for.	AGAGCAATGCAACGTGCAGA	
rev.	TTTGGCGGCTACTTCGCCTA	
CD133		60
for.	CAGAAGGCATATGAATCC	
rev.	CACCACATTTGTTACAGC	
CD44		60
for.	AGCACAATCCAGGCAACTCC	
rev.	CTGGTATGAGCTGAGGCTGC	
SOX2		60
for.	ACCGGCGGCAACCAGAAGAACAG	
rev.	GCGCCGCGGCCGGTATTTAT	
GAPDH		60
for.	TCACCACCATGGAGAAGGC	
rev.	GCTAAGCAGTTGGTGGTGCA	
RPS13		60
for.	CGAAAGCATCTTGAGAGGAACA	
rev.	TCGAGCCAAACGGTGAATC	

for.: forward; rev.: reverse.

## Data Availability

Not applicable.

## Supplementary Materials

The following supporting information can be downloaded at: https://www.mdpi.com/article/10.3390/cancers14092211/s1, Figure S1: MTT assay in different passaged RG cells. Figure S2: Expression of MMR genes in RG cells after ATMi treatment. Figure S3: Cell cycle of RG cells with sub-G1 population after TMZ treatment. Figure S4: Expression of stem markers in LN229-RG cells, Figure S5: Original immunoblots for Figure 6C.
