# Supplementary material for: Characterization of Temozolomide Resistance Using a Novel Acquired Resistance Model in Glioblastoma Cell Lines
Source: Cancers (Basel). 2022 Apr 28;14(9):2211. doi: 10.3390/cancers14092211 (PMC9101568; doi:10.3390/cancers14092211)
Supplement: Supplementary file 1 [file cancers-14-02211-s001.zip › cancers-1650450-supplementary.pdf]

Supplementary Files

Characterization of Temozolomide Resistance Using a Novel Acquired Resistance Model in Glioblastoma Cell Lines

Yuan Zhu, Zhen Chen, Su Na Kim, Chao Gan, Tatsiana Ryl, Michaela Silvia Lesjak, Jan Rodemerk, Rong De Zhong, Karsten Wrede, Philipp Dammann and Ulrich Sure

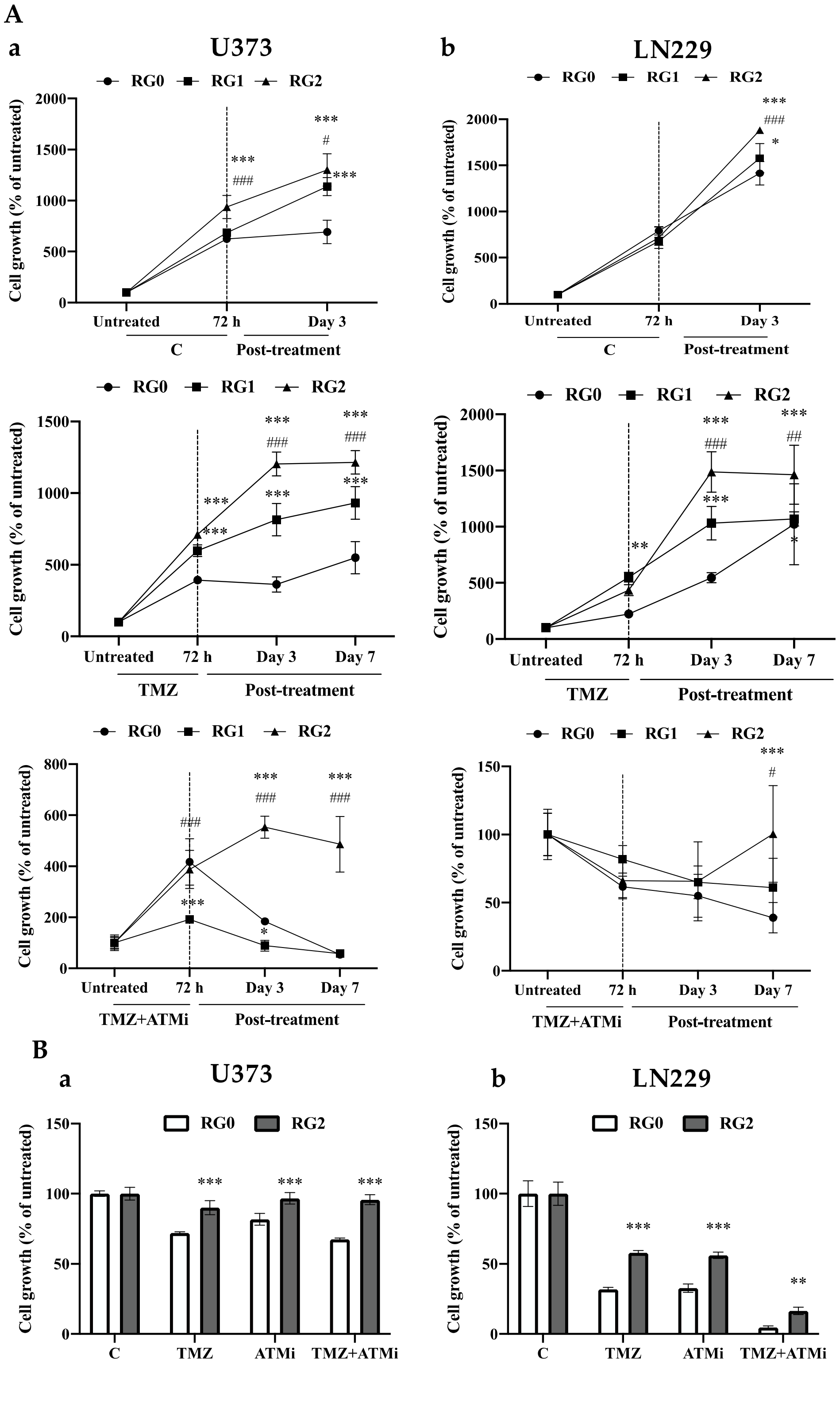

Figure S1. MTT assay in different passaged RG cells. (**A**) U373 RG cells (**a**) and LN229 RG cells (**b**) at passage 3 received the treatment with 0.2% DMSO as vehicle control (C), or TMZ (150 μM), or TMZ+ATMi for 72 h. Thereafter, drugs and dead cells were removed by washing out with fresh medium. The viable cells were cultured for different periods (post-treatment) as indicated followed by MTT assay. (**B**) U373 RG cells (**a**) and LN229 RG cells (**b**) at passage 6 received the treatment with 0.2% DMSO as vehicle control (C) or TMZ (150 μM) or ATMi (10 μM) or TMZ+ATMi for 72 h followed by MTT assay. *, $p < 0.05$; **, $p < 0.01$; ***, $p < 0.001$, compared with RG0; #, $p < 0.05$; ##, $p < 0.01$; ###, $p < 0.001$, compared with RG1.

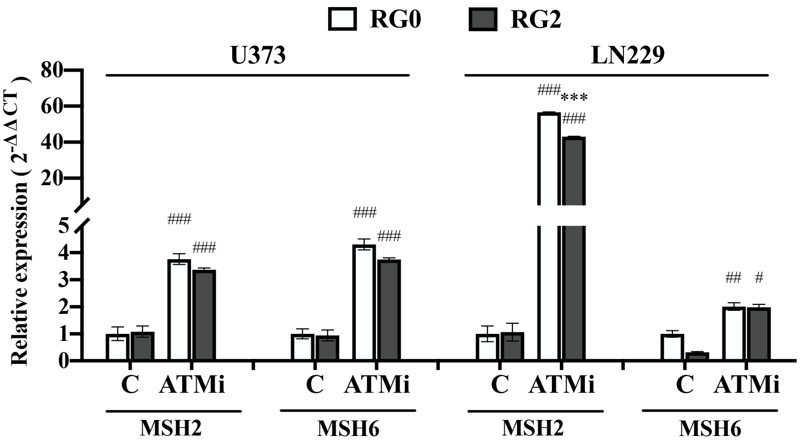

Figure S2. Expression of MMR genes in RG cells. Cells were treated with vehicle (0.2 % DMSO) or with ATMi (10 μM) for 72 h . ***, $p < 0.001$, compared with RG0. #, $p < 0.05$, ##, $p < 0.01$; ###, $p < 0.001$, compared with a corresponding vehicle control (C).

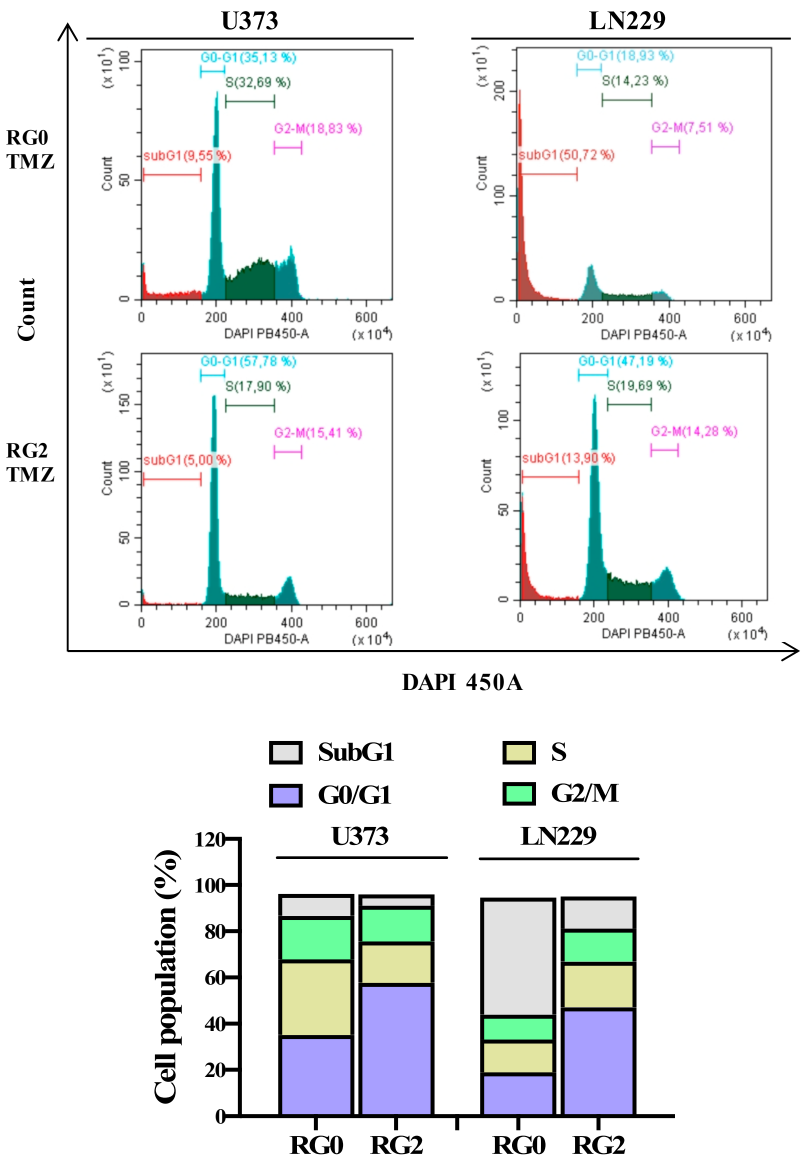

Figure S3. Flow cytometry-based cell cycle assay of RG cells including subG1 phase after 72 h TMZ treatment. In the subG1 phase, RG0 cells exhibited pronounced cell death compared to RG2 cells. Stacked bar graph summarizes the percentage of cells in the cell cycle phases including the subG1 in the experimental groups in the upper panel.

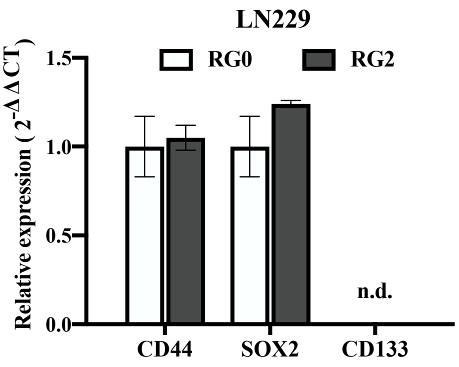

Figure S4. The expression of stem cell markers in LN229-RG cells detected by RT^2-PCR. There was no significant difference in *CD44* and *SOX2* expression between RG0 and RG2. *CD133* expression was not detectable (n.d.) in both RG0 and RG2.

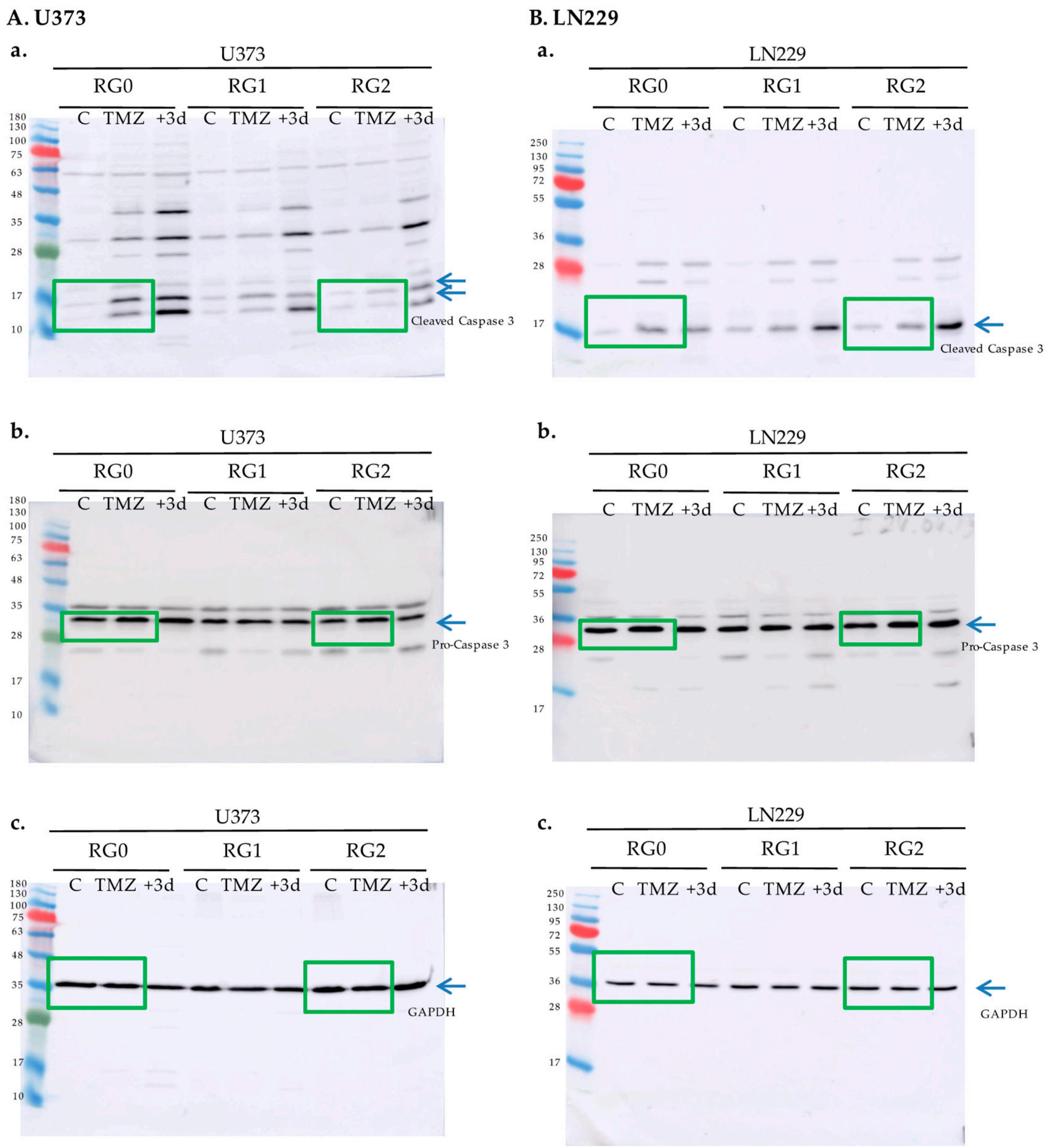

Figure S5. Original immunoblots for Fig. 6C. U373-RGs (**A**) and LN229-RGs (**B**) cells were collected 72 h after treatment with 150 µM of TMZ or vehicle (C) for western blot. Another subset of regrown cells was harvested on day 3 of post treatment, whose data were not included in Fig. 6C. The blots shown in Fig. 6C for cleaved caspase 3 (**a**), pro-caspase 3 (**b**) and GAPDH (**c**) were marked in boxes.
